# Serological investigation and genotyping of *Mycobacterium avium* subsp. *paratuberculosis* in sheep and goats in Inner Mongolia, China

**DOI:** 10.1371/journal.pone.0256628

**Published:** 2021-09-07

**Authors:** Li Zhao, Yu Wang, Jin-Ling Wang, Wei-Hong Zhao, Hui-Xin Cheng, Yi-Min Ma, Hai-Liang Chai, Zhan-Sheng Zhang, Li-Feng Wang, Zeng-Qiang Miao, Yu-Lin Ding, Jirintai Sulijid, Guang-Hui Dang, Shu-Ying Liu, Feng-Long Wang, Si-Guo Liu, Yong-Hong Liu

**Affiliations:** 1 College of Veterinary Medicine, Inner Mongolia Agricultural University, Hohhot, China; 2 Key Laboratory of Clinical Diagnosis and Treatment Technology in Animal Disease, Ministry of Agriculture and Rural Affairs, Hohhot, China; 3 State Key Laboratory of Veterinary Biotechnology, Harbin Veterinary Research Institute, Chinese Academy of Agricultural Sciences, Harbin, China; Cornell University, UNITED STATES

## Abstract

Paratuberculosis a contagious and chronic disease in domestic and wild ruminants, is caused by *Mycobacterium avium* subspecies *paratuberculosis* (MAP). Typical clinical signs include intractable diarrhea, progressive emaciation, proliferative enteropathy, and mesenteric lymphadenitis. Paratuberculosis is endemic to many parts of the world and responsible for considerable economic losses. In this study, different types of paratuberculosis and MAP in sheep and goats were investigated in Inner Mongolia, a northern province in China contiguous with two countries and eight other provinces. A total of 4434 serum samples were collected from six cities in the western, central, and eastern regions of Inner Mongolia and analyzed using the ELISA test. In addition, tissue samples were collected from seven animals that were suspected to be infected with MAP. Finally, these tissues samples were analyzed by histopathological examination followed by polymerase chain reaction (PCR), IS1311 PCR-restriction enzyme analysis (PCR-REA), and a sequence analysis of five genes. Among all 4434 ruminant serum samples collected from the six cities in the western, central, and eastern regions of Inner Mongolia, 7.60% (337/4434) measured positive for the MAP antibody. The proportions of positive MAP antibody results for serum samples collected in the western, central, and eastern regions were 5.10% (105/2058), 6.63% (85/1282), and 13.44% (147/1094), respectively. For the seven suspected infected animals selected from the herd with the highest rate of positivity, the gross pathology and histopathology of the necropsied animals were found to be consistent with the pathological features of paratuberculosis. The PCR analysis further confirmed the diagnosis of paratuberculosis. The rest of the results demonstrated that herds of sheep and goats in Inner Mongolia were infected with both MAP type II and type III. To the best of our knowledge, this is the first study of the two subtypes of MAP strains in sheep and goats in Inner Mongolia.

## Background

*Mycobacterium avium* subsp. *paratuberculosis* (MAP) is a member of the *Mycobacterium avium* complex (MAC) and the causative agent of paratuberculosis (Johne’s disease). The infection primarily affects ruminants and the main signs of infection include diarrhea and wasting. This disease is endemic to many different counties and responsible for considerable economic losses [1–3].

Although MAP can infect a wide range of hosts [3–5], clinical disease has only been reported among ruminants [6, 7], camelids [3, 8], rabbits [9], and hares [10]. Conversely, extensive asymptomatic infections have been observed in non-human primates [11], non-ruminant wildlife [12], dogs [13], feral cats [14], rabbits [15], parrots [16], and bears [17]. Occasional asymptomatic infections have also been found in wild animals [18]. In addition, although it remains controversial whether MAP is the causative agent of Crohn’s disease [3, 19], the presence of MAP in the food chain has been widely acknowledged and has received substantial attention in food industries. The rising awareness of MAP in the food chain has also prompted measures for on-farm control of Johne’s disease.

The capability to discriminate between different MAP strain types has been enhanced by the development of diverse genetic techniques such as epidemiologic analysis, phenotypic characteristics, pulsed-field gel electrophoresis, and IS1311, as well as the recent advances in whole-genome sequence analyses. Currently, MAP strains are divided into two main groups: type C (also designated as type II) and type S. Type C also includes type B, which can be further subdivided into the “Indian Bison type” and “USA Bison type.” Type S can be further subdivided into sub-group types I and III and sub-lineages of camelid isolates [1, 3, 20, 21].

The genotyping of MAP can be used in the study of population genetics, pathogenesis, and molecular epidemiology of paratuberculosis including disease surveillance and outbreak investigation. It can also be used to reveal the transmission of MAP between different species to formulate appropriate policies for disease prevention [1].

## Methods

### Sample collection

This study was carried out in strict accordance with international standards as published in the “Guide to the feeding, management and use of experimental animals” (8th Edition) and follows the “Regulations on the management of experimental animals” and other relevant laws and regulations. The biomedical research ethics committee of Inner Mongolia Agricultural University specifically approved this study (No. 2020[078]). In addition, permission was obtained from the farm owners before the specimens were collected, and all efforts were made to minimize suffering.

Serum samples from a total of 4434 animals (sheep and goats) were collected from six cities (A–F) in the western, central, and eastern regions of Inner Mongolia during August 2018 and September 2019. The tested ruminants were selected randomly. However, the sampling process did not exactly follow the original design owing to certain restrictions. Specifically, the serum samples were collected from each region at the same time as the antibody tests that were conducted after the epidemic prevention action in the spring and fall of each year. The samples were taken from sheep and goats (mixed herds) of all ages in both centralized and individual farms. These serum samples were then tested to measure the status of MAP infection in each animal. Based on the test information, the herd with the highest rate of positivity of the MAP antibody (i.e., the highest rate of antibodies) was identified. The sick sheep and goats (showing diarrhea and wasting) were monitored until they died of the disease. Afterward, the intestine and mesenteric lymph node tissue samples were collected from the seven dead animals (Table 1). In particular, two sets of samples were collected from each animal, one set was fixed in 10% neutral formalin and the other set was preserved by freezing.

**Table 1:** Case information of sheep and goats suspected to be infected with MAP.

Case No.	Case in Inner Mongolia	Species	Sex	Age	Source	Frozen tissue samples	Altitude (m), Latitude and Longitude
1	Eastern Region	Goat	Female	Over one year old	Individual farmer	Intestines	209; 44°40′N, 121°33′E
2	Eastern Region	Goat	Female	Over one year old	Farm	Intestines	653; 42°37′N, 119°83′E
3	Western Region	Goat	Female	Nine months old	Individual farmer	Mesenteric lymph nodes	1008; 40°58′N, 110°55′E
4	Eastern Region	Sheep	Female	Over one year old	Individual farmer	Mesenteric lymph nodes	252; 47°23′N, 122°50′E
5	Western Region	Goat	Female	Over one year old	Farm	Intestines	1267; 38°29′N, 108°74′E
6	Central region	Sheep	Female	Over one year old	Individual farmer	Mesenteric lymph nodes	1016; 40°64′N, 111°56′E
7	Western Region	Goat	Female	Over one year old	Individual farmer	Intestines	1226; 41°09′N, 105°43′E

### Detection of MAP antibody

The MAP antibodies in the 4434 serum samples were measured using a *Mycobacterium paratuberculosis* Antibody Test Kit (Paratuberculosis Screening) (IDEXX, France, Code No. 06-07130-27) following the manufacturer’s protocol. Sera with S/P ratios ≤ 0.45 and ≥ 0.55 were considered negative and positive, respectively. Intermediate S/P values were considered “suspect” and regarded as negative for the data analysis.

### Pathological examination

Paraffin-embedded pathological tissue sections were first obtained by the standard tissue processing from the fixation of intestine and mesenteric lymph nodes in 10% neutral formalin. These tissue sections were then used for histopathological observation and the acid-fast bacilli test after hematoxylin and eosin staining and Ziehl–Neelsen staining.

### DNA extraction, polymerase chain reaction (PCR) amplification, enzyme digestion, DNA sequencing, and sequence analysis

The genomic DNA were extracted from the seven cryopreserved tissue samples using a Ezup Column Animal Genomic DNA Purification Kit (Sangon Biotech, Shanghai, China, Code No. B518251) following the manufacturer’s instructions. All the extracted DNA samples were stored at -20°C for later usage.

One microliter of extracted DNA template was used for PCR amplification. The PCR was performed using Taq PCR Master Mix (2X, with Blue Dye) (Sangon Biotech, Shanghai, China, Code No. B639295) with primers specific for IS900 [22], DMC [23], IS1311 [24], F57 [25], and 16S rRNA [22]. The PCR products from the amplification of IS1311 were digested with HinfI and MseI. Finally, 5 μL of PCR product was analyzed by electrophoresis using 1.5% agarose gel.

All the samples that tested positive were sent to a commercial company (Sangon Biotech, Shanghai, China) for sequence analysis. The sequences were aligned with reference sequences downloaded from GenBank using MEGA 5.0 software. The sequencing results were analyzed using the BLAST online platform. To assess the phylogenetic relationships of MAP IS900 gene sequences obtained in this study and those downloaded from GenBank, phylogenetic trees were constructed using the neighbor-joining algorithm, based on a matrix of evolutionary distances calculated using the Kimura 2-parameter model by the MEGA 5.0 software. Bootstrap analysis was used to assess the robustness of clusters using 500 replicates.

## Results

### Survey of the sera

Among all 4434 ruminant serum samples collected from the six cities in the western, central, and eastern regions of Inner Mongolia from 2018 to 2019, 7.60% (337/4434) tested positive for the MAP antibody. The percentage of positive MAP antibody results for serum samples collected in the western, central, and eastern regions were 5.10% (105/2058), 6.63% (85/1282), and 13.44% (147/1094), respectively. The percent positivity rate in cities A–F were 5.19%, 4.84%, 6.23%, 7.23%, 11.64%, and 14.76%, respectively (Table 2).

**Table 2:** Detection of the MAP antibody in ruminants (sheep and goats) in Inner Mongolia.

Sampling sites in Inner Mongolia		Sampling No.	Number of positive samples	Positive rate (%)
Western Region	A1	220	8	3.64
	A2	320	13	4.06
	A3	180	5	2.78
	A4	186	9	4.84
	A5	150	8	5.33
	A6	150	0	0
	A7	196	24	12.24
	A8	160	14	8.75
	Total number of A	1562	81	5.19
	B	496	24	4.84
Central region	C	770	48	6.23
	D	512	37	7.23
Eastern Region	E	464	54	11.64
	F	630	93	14.76
Total number of samples		4434	337	7.60

### Pathological changes in the tissues

**Fig 1:**
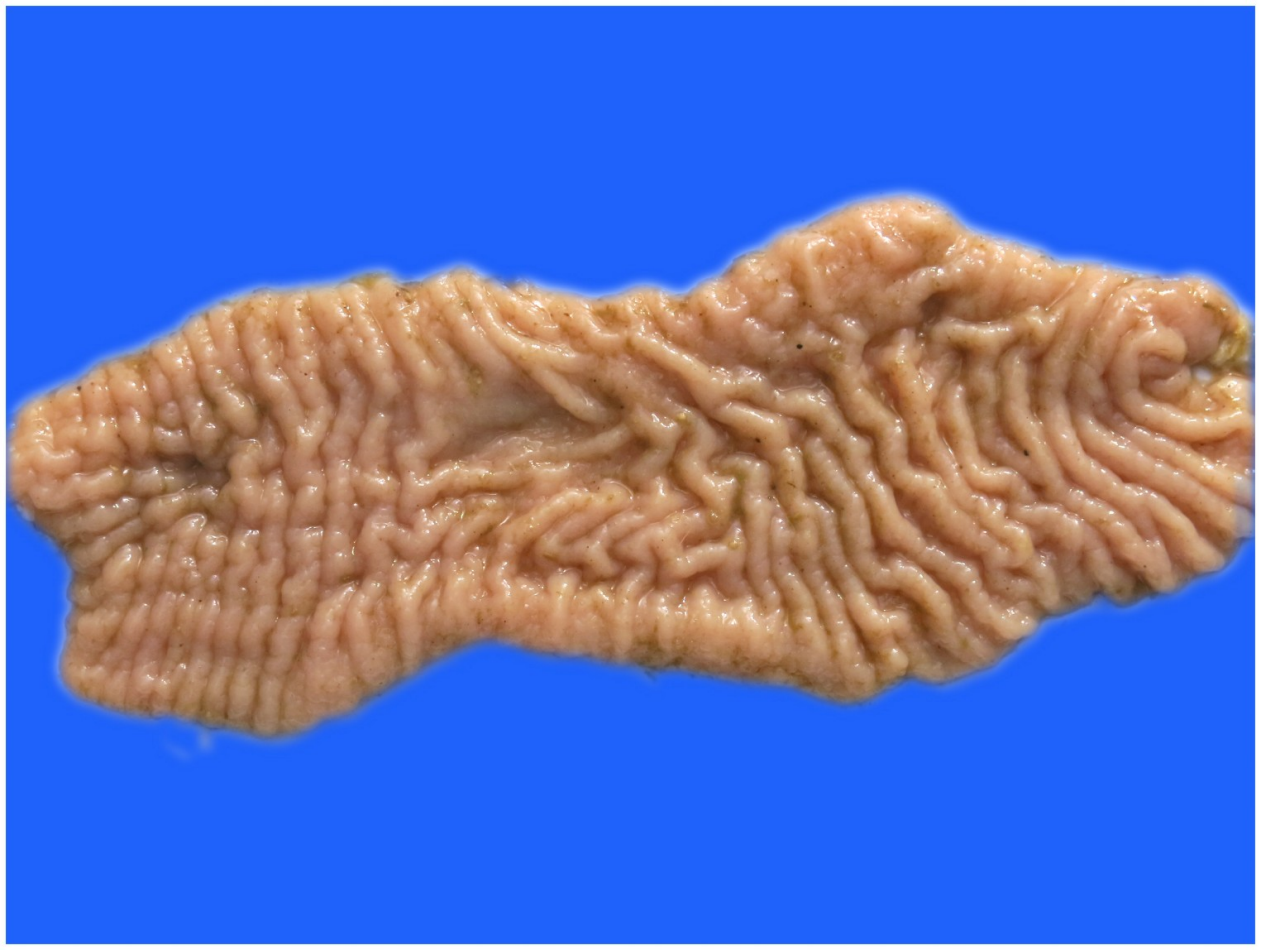
Bowel wall thickening and mucosal folding in the cecum.

**Fig 2:**
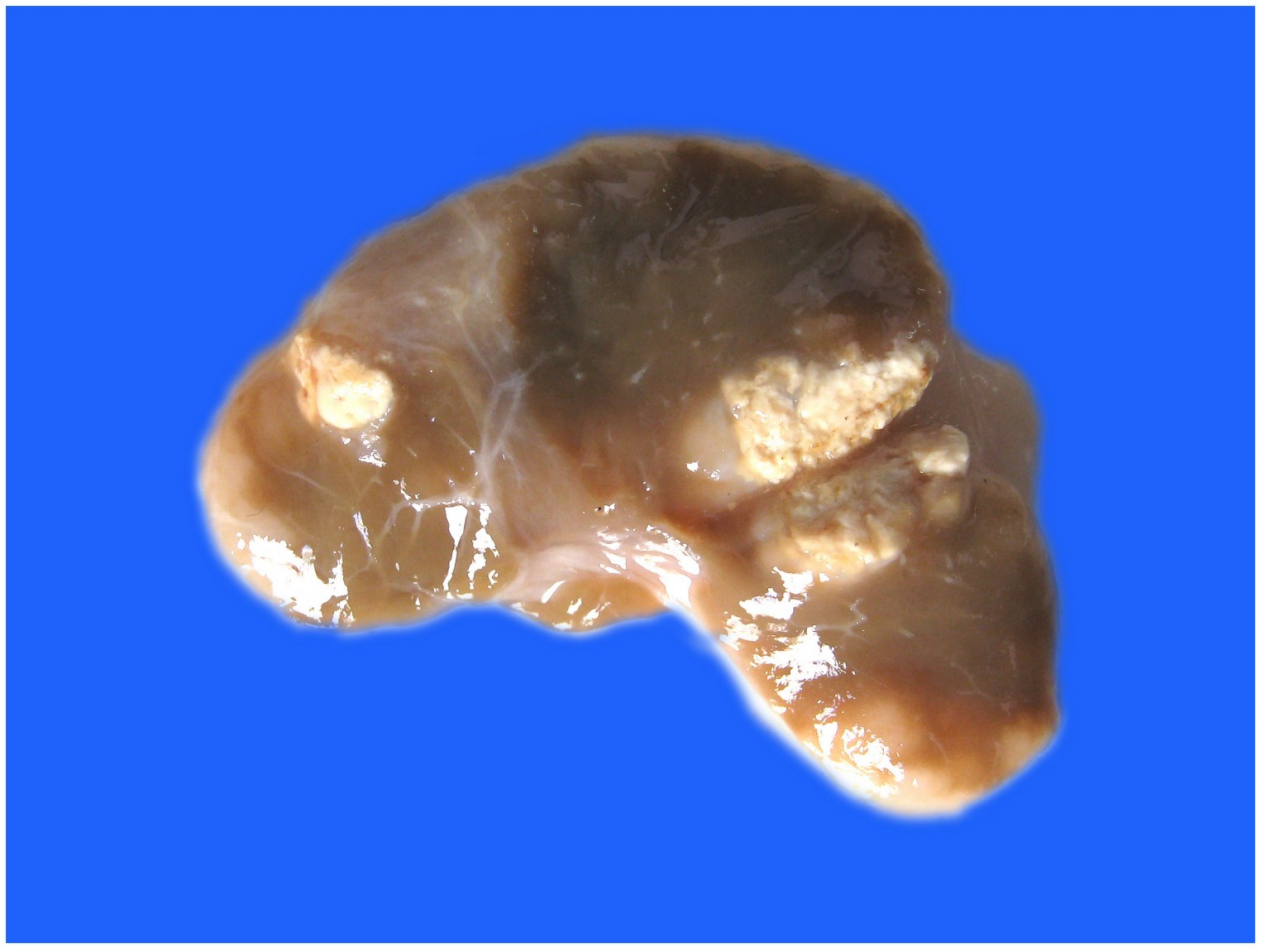
Inflammation of mesenteric lymph nodes; grayish-white lesions of different sizes were observed on the surface and the cut section.

**Fig 3:**
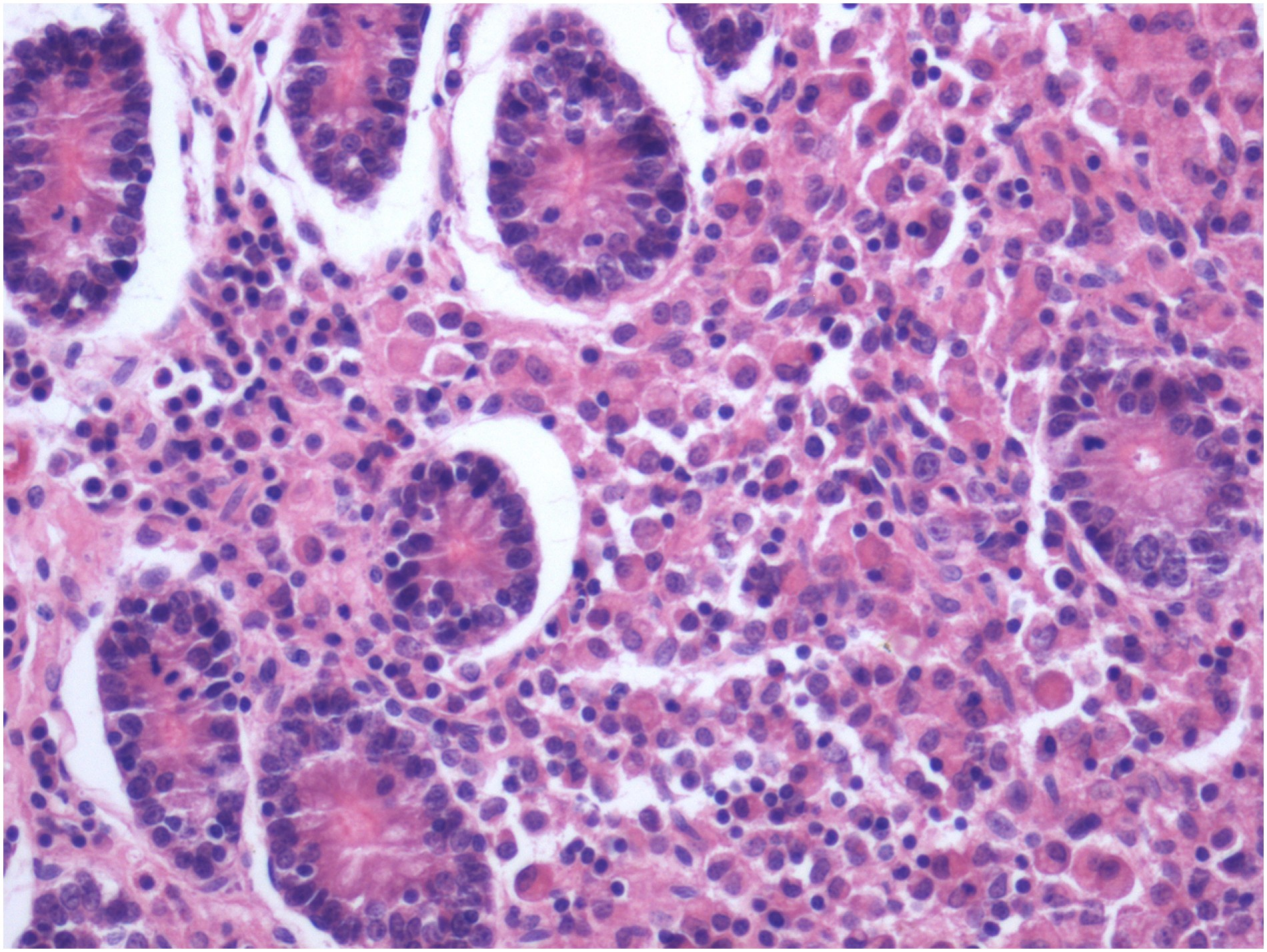
Proliferation of epithelioid cells and lymphocytes in ileum propria (hematoxylin and eosin, ×200).

**Fig 4:**
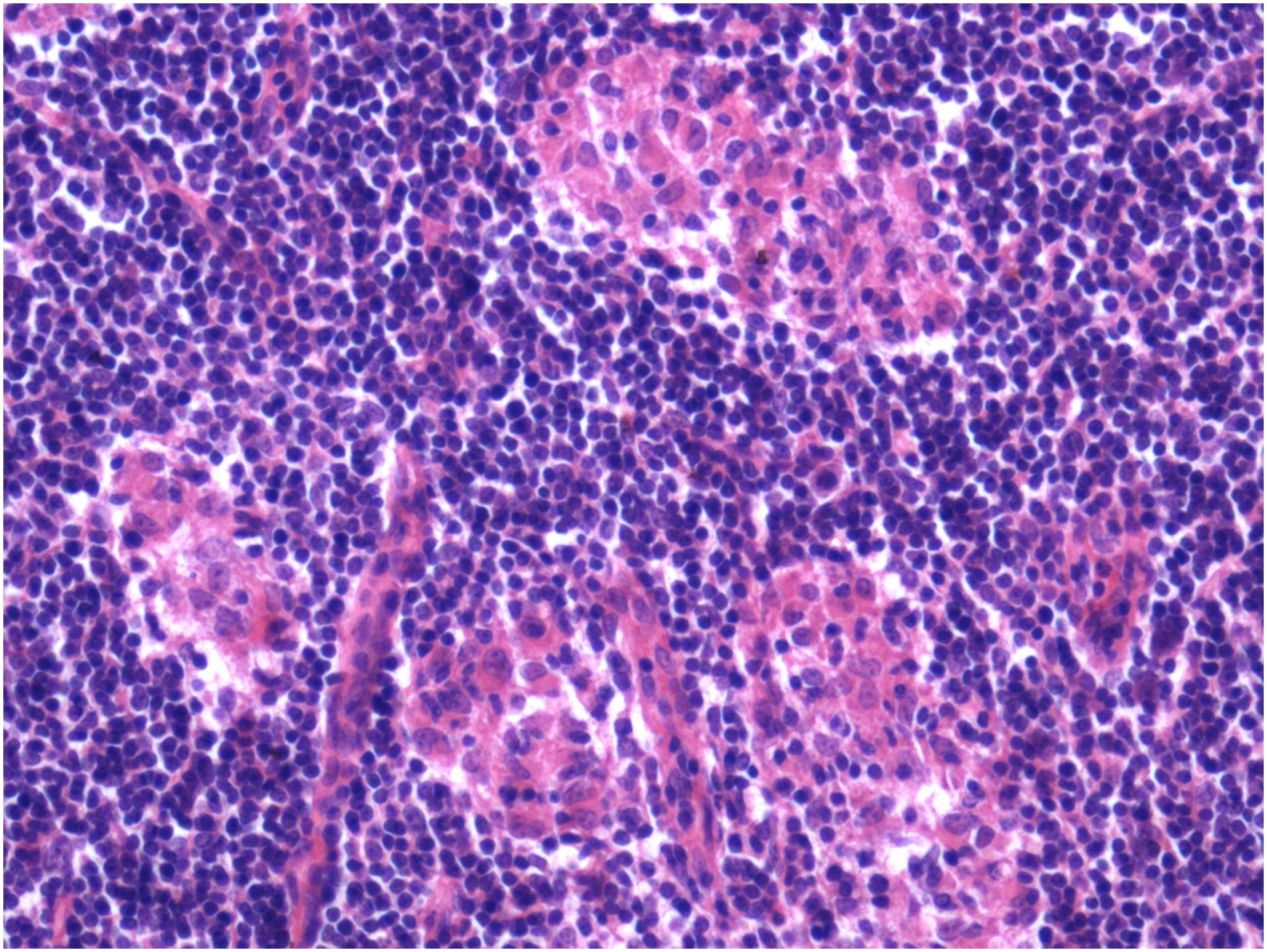
Epithelial hyperplasias in the cortex of the lymph node (hematoxylin and eosin, ×100).

**Fig 5:**
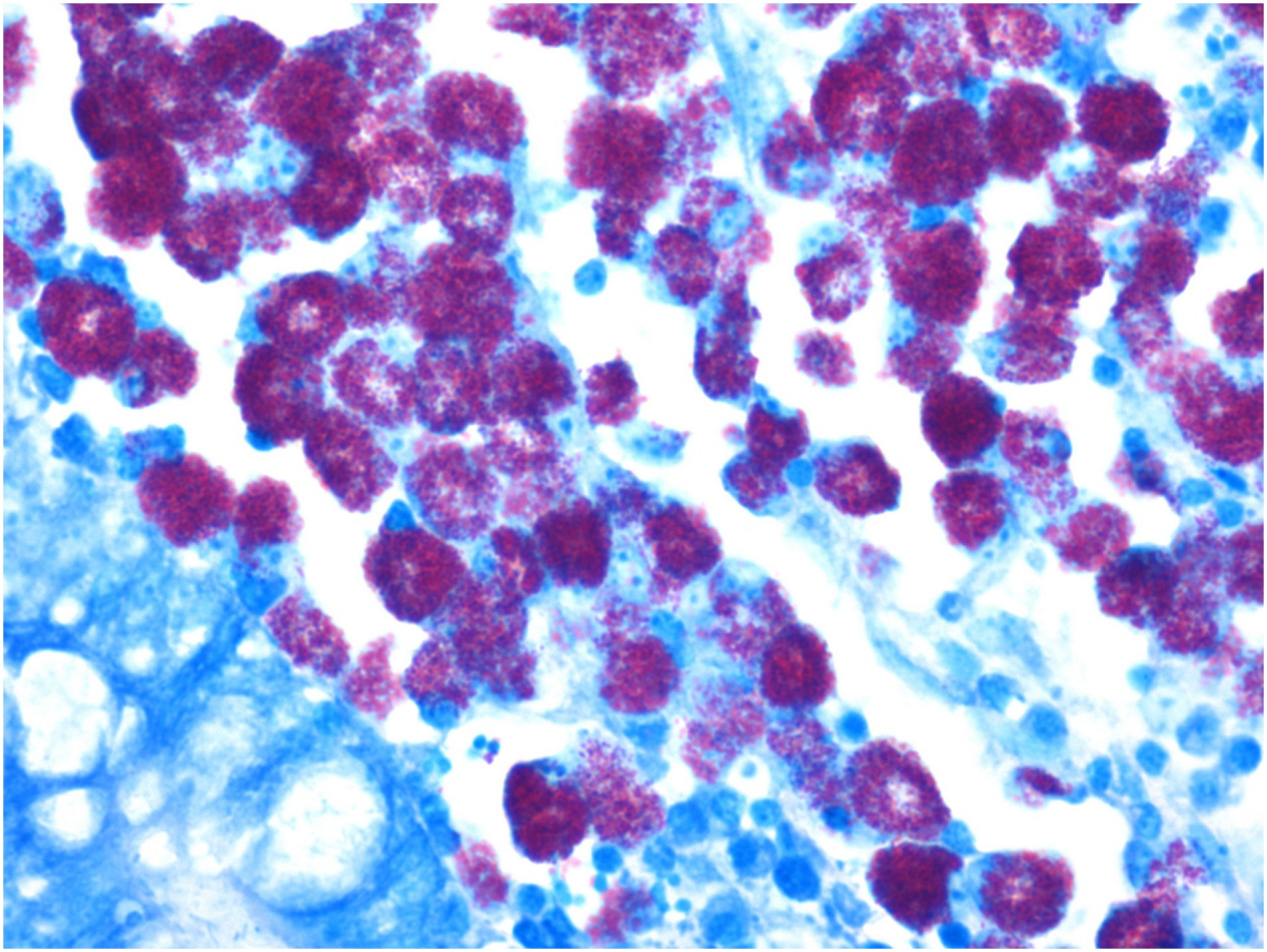
A significant number of acid-fast bacilli were observed in the cytoplasm of epithelioid cells in cecum propria (Ziehl–Neelsen, ×400).

Visual observation of tissues obtained from the carcasses revealed dilatation of the intestine, narrowing of the center opening of the intestine, and thickening of the bowel wall (Fig 1). In addition, the mesenteric lymph nodes had become swollen, pale, discolored, and had hardened. Grayish-white cheese-like lesions were observed on the surface and the cut section (Fig 2). Observation under the microscope revealed a large number of epithelioid cells and lymphocyte proliferation in the lamina propria of the intestinal mucosa (Fig 3) without significant necrosis or intestinal gland atrophy. The proliferation of substantial epithelioid cells in the mesenteric lymph node cortex and lymph sinuses led to the formation of epithelioid cell nodules (Fig 4). Significant proliferation of lymphocytes was observed in the entire lymph node and found to be the most prominent in the paracortical area. Proliferated epithelioid cells containing a large number of acid-fast bacilli were found in Ziehl–Neelsen-stained intestinal tissue and mesenteric lymph nodes (Fig 5).

### Identification and strain typing of MAP and sequence analysis

#### Results of PCR amplification and enzyme digestion

**Fig 6:**
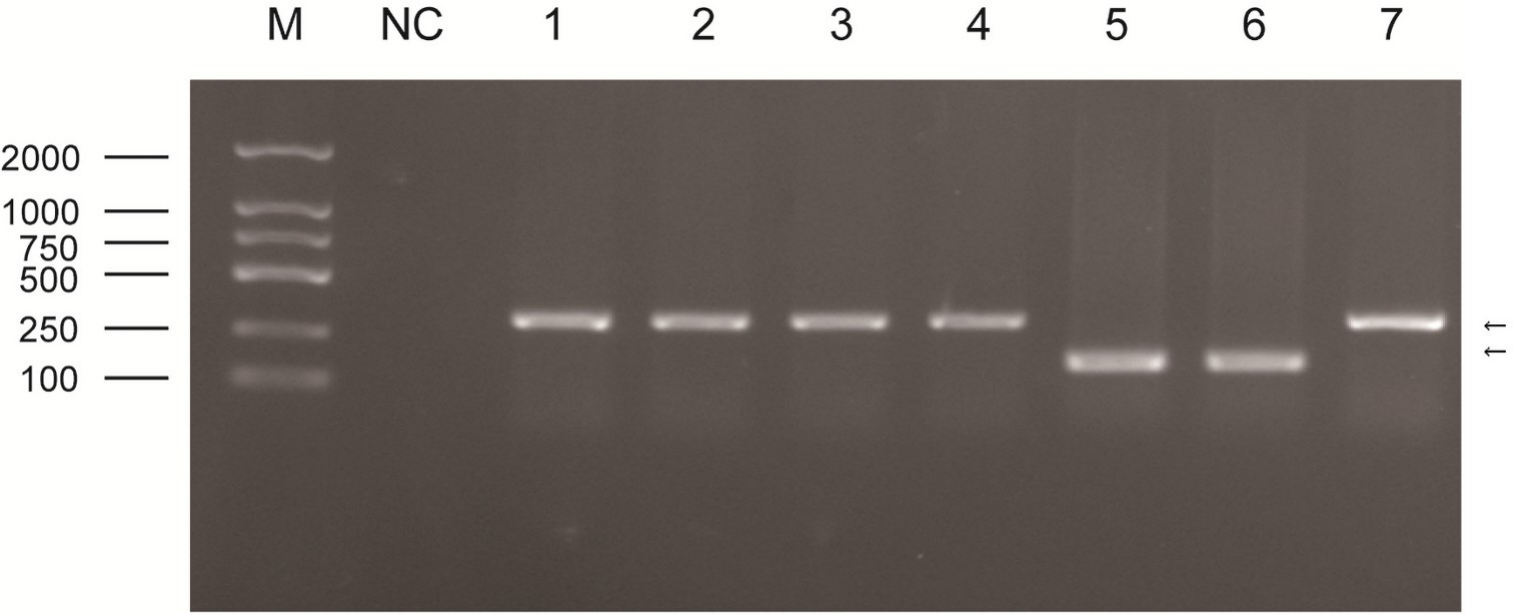
PCR results of seven pathological tissue samples obtained from the amplification of the DMC gene (M: Maker; NC: Negative control; 1 to 4: Case-1 to Case-4; 5 to 6: Case-6 and Case-7; 7: Case-5).

**Fig 7:**
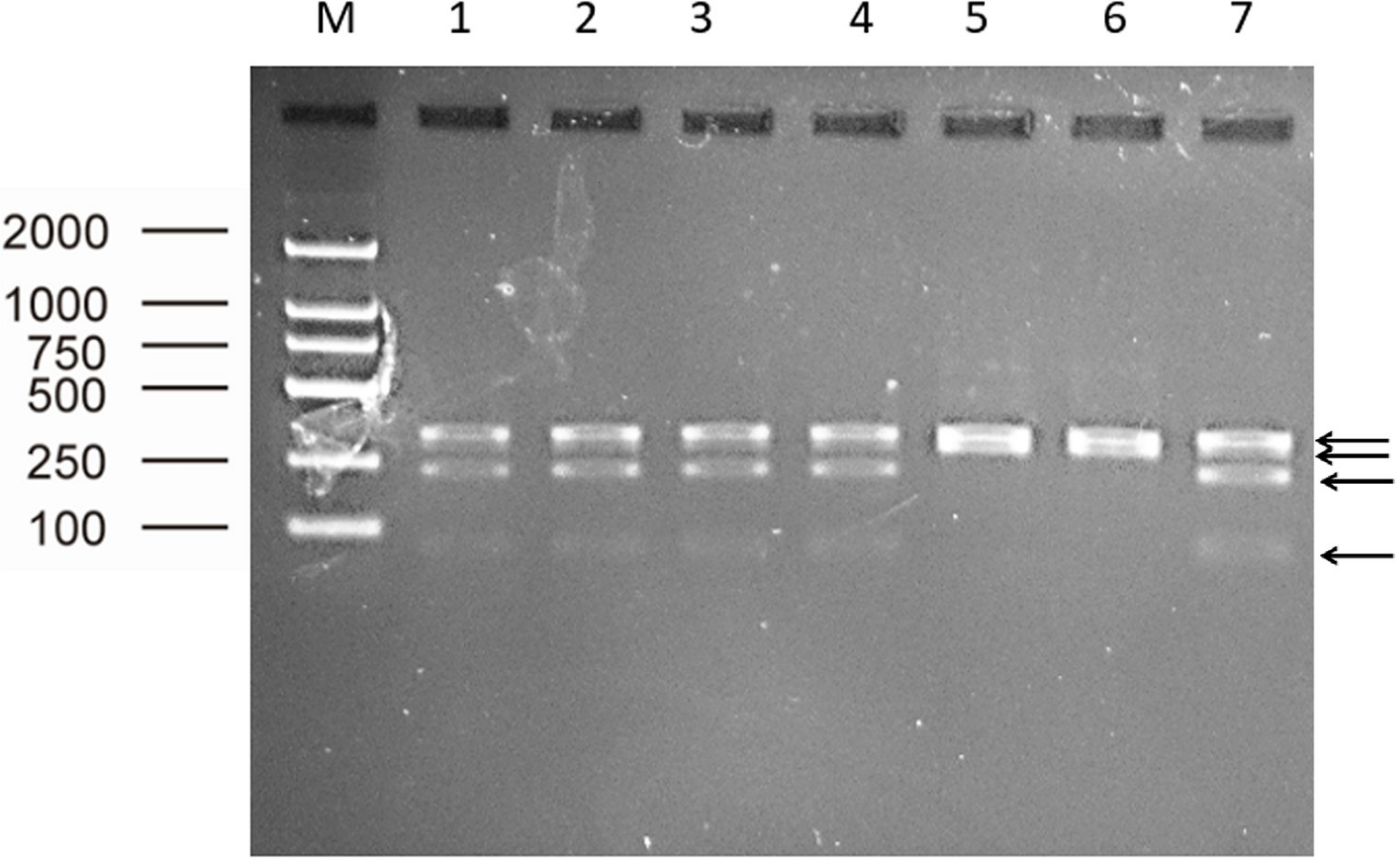
PCR-REA results of seven pathological tissue samples (M: Maker; 1 to 4: Case-1 to Case-4; 5 to 6: Case-6 and Case-7; 7: Case-5).

The PCR amplification products of the seven pathological tissue samples obtained using five primers (specific for IS900, DMC, IS1311, F57, and 16S rRNA) all tested positive. The sizes of the amplification products were consistent with our expectations. Specifically, the size of the PCR products of Case-1 to Case-5 DMC genes was approximately 310 bp. The size of the PCR products of Case-6 to Case-7 genes was approximately 162 bp (Fig 6). The size of the PCR products of the seven pathological tissue samples obtained from the amplification of IS1311 was approximately 608 bp. After digestion with HinfI and MseI, four stripes of PCR products (with sizes of 67 bp, 218 bp, 285 bp, and 323 bp) were observed for Case-1 to Case-5, whereas two stripes of PCR products (285 bp and 323 bp) were found in Case-6 and Case-7 (Fig 7).

#### Sequence analysis results of the IS900 gene

The seven IS900 gene segments obtained in this study are only identical to the original IS900 sequence (MAP strain Ben, GenBank accession no. X16293). The guanine–cytosine (GC) base pair was missing at positions 36 and 37 in the IS900 gene compared to the rest of the reference strains explored in this study, such as MAP strain K-10 (the strain name, GenBank accession number, and MAP type information can be found in the phylogenetic nomenclature for the strains). In addition, the following features were found when comparing the seven IS900 gene segments obtained in this study with all the MAP reference strains investigated: (1) the MAP strain Ben at positions 122 and 123 in the IS900 gene was converted from a GC base pair to a CG base pair; (2) thymine (T) was observed at position 169 in the IS900 gene for Case-6 and Case-7, but cytosine (C) was observed at the same position for Case-1 to Case-5; (3) guanine (G) was observed at position 216 in the IS900 gene for Case-6 and Case-7, whereas adenine (A) was observed at the same position for Case-1 to Case-5; (4) a distinctive change was observed in the IS900 gene for Case-5 (different from all the reference strains) in which the G at position 268 was converted to C.

#### Sequence analysis results of the IS1311 gene

The seven IS1311 gene segments obtained in this study were analyzed and compared with the following five strains at positions 64, 65, 68, 223, 236, 422, 527, and 628: (1) the representative strain of Indian Bison type ‘Bison type’ S5 (EF514838); (2) the representative strain of type C (cattle) 316V (AJ223974); (3) the representative strain of type S (sheep) JD97/1-2 (AJ223975); (4) the representative strain of USA Bison type 98/1010 (AJ308375); and (5) *M*. *avium* subsp. *avium* (MAA) strain IMM147460 (U16276). The results demonstrated that Case-1 to Case-5 might be type B or type C, whereas Case-6 and Case-7 might be type S or type C (Table 3).

**Table 3:** Results of the multipoint analysis of the IS1311 gene for Case-1 to Case-7.

Strain	Species and Type	Position on IS1311						
		64, 65	68	223	236	422	527	628
Case-1 to Case-5	This study	TG	C	T	C	C	A	N
Case-6 and Case-7	This study	TG	C	C	C	C	A	N
316V (AJ223974)	MAP, type C	TG	C	C or T	C	C	A	C
JD97/1-2 (AJ223975)	MAP, type S	TG	C	C	C	C	A	C
98/1010 (AJ308375)	MAP, USA Bison type	TG	C	T	C	C	A	C
’Bison type’ S5 (EF514838)	MAP, Indian Bison type	--	C	T	C	N	N	N
IMM147460 (U16276)	MAA	TG	T	C	T	T	G	T

Note: N stands for uncertainty

#### BLAST sequence analysis results

A BLAST sequence analysis was performed on the seven IS900 gene segments, the seven DMC gene segments, and the seven IS1311 gene segments obtained in this study. As indicated by the values of per. ident, total score, and max score in the BLAST results, the strain that shares the highest similarity with Case-6 and Case-7 is type III, whereas the strain that shares the highest similarity with Case-1 to Case-5 is type II. However, the BLAST analysis of the seven F57 gene segments obtained in this study show that they share consistent features with type I, type II, and multiple strains in type III. Therefore, the MAP type could not be determined by the F57 gene. Finally, the seven 16S rRNA obtained in this study were shown to share an identical gene sequence. However, the BLAST analysis indicated that these rRNAs also share identical features with MAA, *M*. *avium* subsp. *hominissuis* (MAH), and multiple strains of MAP. Therefore, the MAP species could not be determined by the 16S rRNA (Table 4).

**Table 4:** BLAST comparison analysis results of the MAP IS900, DMC, IS1311, F57, and 16S rRNA genes.

Case No.	Name of the gene	Name of the most homologous sequence	GenBank accession number	Nucleotide homology (%)	Remarks (type, origin)
1	IS900	DSM 44135	CP053068	99.50	Type II, Germany
	DSM	DSM 44135	CP053068	99.63	Type II, Germany
	IS1311	MAPK_CN7/15	CP033428	99.48	Type II, South Korea
	F57	DSM 44135	CP053068	98.00	Type II, Germany
		Telford	CP033688	98.00	Type I, Australia
		JIII-386	CP042454	98.00	Type III, Germany
2	IS900	DSM 44135	CP053068	99.25	Type II, Germany
	DSM	DSM 44135	CP053068	100	Type II, Germany
	IS1311	MAPK_JJ1/13	CP033909	99.82	Type II, South Korea
	F57	DSM 44135	CP053068	98.00	Type II, Germany
		Telford	CP033688	98.00	Type I, Australia
		JIII-386	CP042454	98.00	Type III, Germany
3	IS900	DSM 44135	CP053068	99.25	Type II, Germany
	DSM	DSM 44135	CP053068	99.63	Type II, Germany
	IS1311	MAPK_CN7/15	CP033428	99.48	Type II, South Korea
	F57	DSM 44135	CP053068	98.25	Type II, Germany
		Telford	CP033688	98.25	Type I, Australia
		JIII-386	CP042454	98.25	Type III, Germany
4	IS900	DSM 44135	CP053068	99.25	Type II, Germany
	DSM	DSM 44135	CP053068	99.28	Type II, Germany
	IS1311	MAPK_CN7/15	CP033428	99.30	Type II, South Korea
	F57	DSM 44135	CP053068	99.00	Type II, Germany
		Telford	CP033688	99.00	Type I, Australia
		JIII-386	CP042454	99.00	Type III, Germany
5	IS900	DSM 44135	CP053068	99.25	Type II, Germany
	DSM	DSM 44135	CP053068	99.28	Type II, Germany
	IS1311	MAPK_CN7/15	CP033428	99.48	Type II, South Korea
	F57	DSM 44135	CP053068	98.25	Type II, Germany
		Telford	CP033688	98.25	Type I, Australia
		JIII-386	CP042454	98.25	Type III, Germany
6	IS900	JIII-386	CP042454	99.50	Type III, Germany
	DSM	JIII-386	CP042454	99.18	Type III, Germany
	IS1311	JIII-386	CP042454	99.82	Type III, Germany
	F57	DSM 44135	CP053068	99.74	Type II, Germany
		Telford	CP033688	99.74	Type I, Australia
		JIII-386	CP042454	99.74	Type III, Germany
7	IS900	JIII-386	CP042454	99.25	Type III, Germany
	DSM	JIII-386	CP042454	98.44	Type III, Germany
	IS1311	JIII-386	CP042454	99.82	Type III, Germany
	F57	DSM 44135	CP053068	98.96	Type II, Germany
		Telford	CP033688	98.96	Type I, Australia
		JIII-386	CP042454	98.96	Type III, Germany
1~7	16S	DSM 44135	CP053068	99.93	MAP
		DSM 44156	CP046507	99.93	MAA
		JP-H-1	AP020326	99.93	MAH

#### Analysis of the phylogenetic tree for the IS900 gene

**Fig 8:**
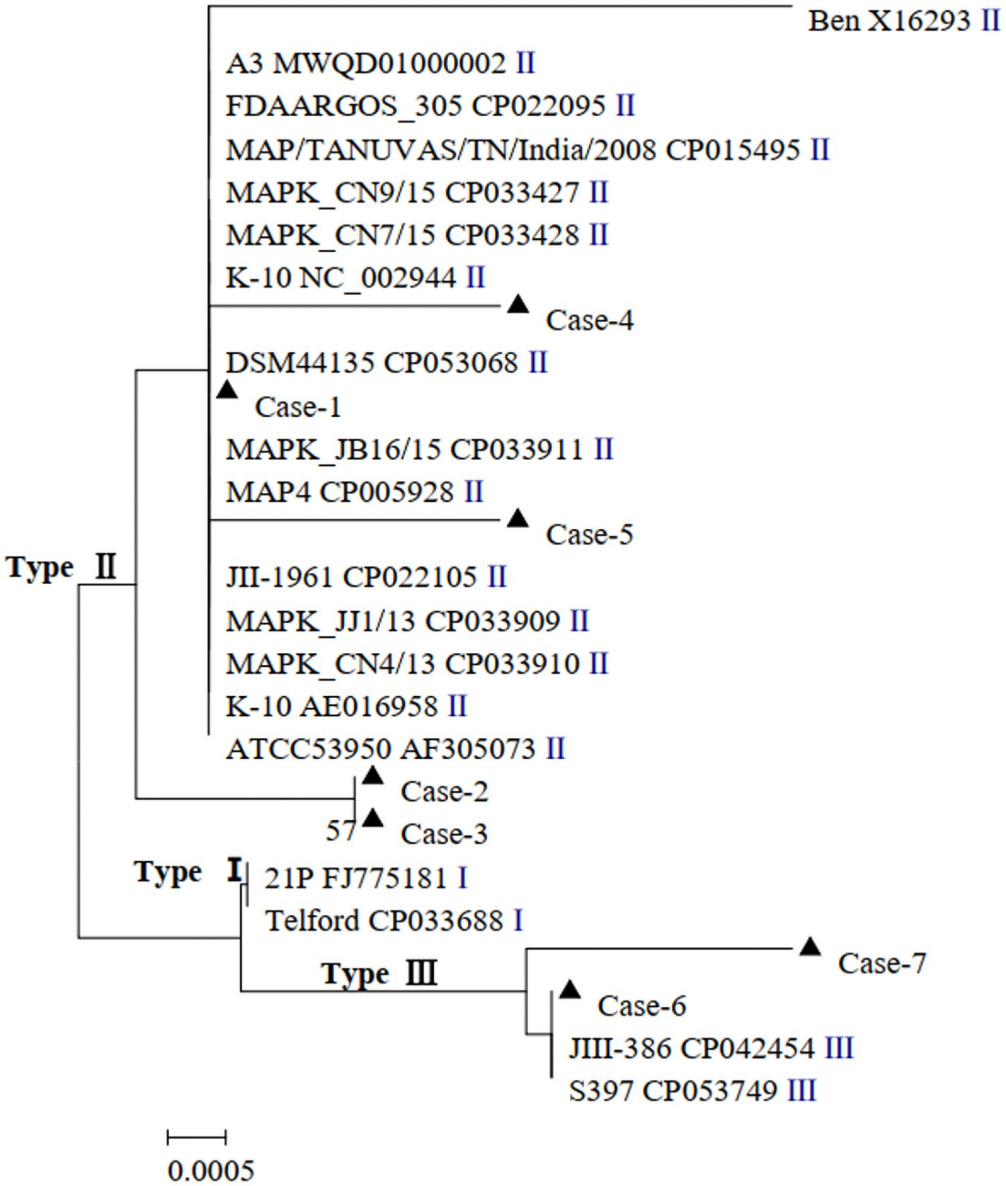
Phylogenetic tree of IS900 gene sequences from MAP. The evolutionary history was inferred using the neighbor-joining method base on the Kimura 2-parameter model. The analysis involved 26 nucleotide sequences. There were a total of 400 positions in the final dataset. Evolutionary analyses were conducted in MEGA5. Sequences of this study were marked with a black triangle (▲).

The 26 nucleotide sequences investigated in this study, including MAP strains type I, type II, and type III sequences reported in previous studies (the sequences are named by Strain+GenBank+Type), were analyzed using the evolutionary history at 400 positions in the IS900 gene using MEGA 5.0. Different types of strains could be grouped under one branch. As shown by the analysis, Case-1 to Case-5 were grouped together with the type II reference strain, whereas Case-6 and Case-7 were grouped together with the type III reference strain (Fig 8).

## Discussion

Paratuberculosis is a major production-limiting disease of livestock. “Wasting or consumptive” disease was first reported in 1807 in cattle and later described as “pseudotuberculous enteritis” by Johne and Frothingham in 1895. In 1923, *Mycobacterium* paratuberculosis was officially named as the pathogen of paratuberculosis [7]. In China, bovine paratuberculosis was first reported to occur in Inner Mongolia in 1953. Later, the occurrence of bovine paratuberculosis was reported in a number of different cities [26]. The occurrence of ovine paratuberculosis in China was first reported in 1971. Subsequently, sheep paratuberculosis was reported to occur in a number of different provinces and cities in China. Currently, MAP infections in sheep and goats have been found in many different countries. Ovine paratuberculosis has been found in the southern hemisphere in New Zealand, Australia, and South Africa; numerous northern hemisphere countries, particularly Norway, Great Britain, and Austria; and Mediterranean countries, including Greece, Portugal, Morocco, Spain, and Jordan. Caprine paratuberculosis has already been diagnosed in Turkey, Norway, France, Switzerland, Canada, Croatia, the USA, and Chile [27]. As shown by the earlier test reports, the prevalence of paratuberculosis in Shandong Province, China in 2011 and 2012 was 29.34% and 14.93%, respectively. The prevalence of paratuberculosis in Tibet, Shanghai, and Guangxi Provinces varies between 2% and 4%. However, a highly variable prevalence (0–73.4%) was reported in Inner Mongolia [26]. In comparison, a similarly high prevalence has also been reported in Europe and other western countries. As shown by reports from 2011 to 2016, in cattle, the high between-herd prevalence (HTP) was found to be 91.1% in the USA, 68–76% in Canada, 87–96% in Denmark, and approximately 70% in Northern Italy. In sheep, the HTP was assessed to be 66.8% in Canada and 76% in New Zealand. An HTP of 62.6% was calculated in dairy goats in Canada, 74.1% in water buffaloes in Italy, and 46% and 42% in New Zealand deer and beef herds, respectively [7]. There are no countries that claim to be free of MAP.

As shown by our survey of MAP antibodies in sheep and goat sera collected from Inner Mongolia, the overall positivity rate was 7.60% (337/4434). In addition, the positivity rate was found to be significantly higher in the eastern region than that in the western region of Inner Mongolia. The low level of serological individual positive rates observed in this study is primarily caused by the long incubation period of paratuberculosis as a chronic disease. If the serum samples had been collected only from mature animals (over 1 yr old), then the test result may yield a higher positivity rate. Because the sheep and goats in Inner Mongolia never receive the paratuberculosis vaccine, the serological data obtained in this study provide strong evidence that paratuberculosis is present in this region. In the subsequent analysis, we selected the herd showed the highest rate of positivity of the MAP antibody. We then monitored seven animals that were suspected to have paratuberculosis in this herd. Finally, the tissue samples from these sheep and goats were collected to analyze their infection status. The pathological examination, multi-gene PCR, and sequence analysis of these samples further confirmed that the sheep and goats in Inner Mongolia were infected with MAP.

The epidemiological information about this disease has been minimal in most of the prevalent regions of the world, except for the USA and a few European countries [28]. There have been very few studies of paratuberculosis in Inner Mongolia or even China as a whole. Furthermore, the majority of the studies in China focused on bovine paratuberculosis through epidemiological and serological surveys. Understanding the genetic variability of MAP strains is important in diagnosis, epidemiological investigation, and the formation of strategies for prevention and control of the disease. Specifically, strain genotyping is a valuable tool for epidemiological tracing of pathogenic microorganisms.

Although the first occurrence of paratuberculosis in China were in Inner Mongolia, there has been no report on MAP genotyping in Inner Mongolia. To further obtain the molecular identification of paratuberculosis and the strain type of MAP, we confirmed the identity of isolates as MAP by using (a) the F57 genetic element, which is unique to MAP and (b) the 16S rRNA gene, which is capable of identifying *Mycobacterium*. Additional strain typing of paratuberculosis was performed using the IS900, IS1311, and DMC genes. As shown by the PCR results of the seven pathological tissue samples obtained from the amplification of five genes, Case-1 to Case-7 MAP all tested positive.

Because the gene sequence of DMC is different in type C and type S MAP, the amplification products obtained using different primers specific for different MAP types (as reported in the literature) will have different sizes. Such a difference allows us to differentiate between type C and type S MAP [23]. The PCR products of Case-1 to Case-5 samples obtained from the amplification of the DMC gene are the same size as the PCR product of type C MAP; the PCR products of Case-6 and Case-7 samples are the same size as the PCR product of type S MAP.

The restriction enzyme digestion of different types of MAP will yield different results owing to the different canonical nucleobases located at Position 223 in the IS1311 gene. Therefore, it is possible to identify type C, type S, or type B MAP from PCR-restriction enzyme analysis (PCR-REA) of the IS1311 gene [1, 24, 29, 30]. After the IS1311 gene PCR products were digested with HinfI and MseI, the number of stripes for Case-1 to Case-5 samples was consistent with that in other reports of type C, whereas the number of stripes for Case-6 and Case-7 samples was consistent with that in other studies of type S.

The IS1311 gene segments obtained in this study were analyzed and compared with different representative MAP strains reported in previous studies [1, 29, 31] at positions 64, 65, 68, 223, 236, 422, 527, and 628. The comparison confirmed that (a) Case-1 to Case-7 are not Indian Bison type, (b) Case-1 to Case-5 might be type B or type C, and (c) Case-6 and Case-7 might be type S or type C.

During the point analysis of the IS900 gene segment, the information at positions 169 and 216 played a dominant role in determining the strain type. Previous studies have shown that (a) type III has a T or C/T at position 169, whereas type I and type II have a C at the same position; (b) type III has a G or A/G at position 216, and type I and type II have a G and A, respectively, at the same position [32, 33]. Consistent results have been obtained in this study using the type III reference strains (S397, JIII-386, and CAM86), type I reference strains (Telford and 21P), and several other type II reference strains. Based on the information at position 169, Case-1 to Case-5 might be type I, type II, or type III, whereas Case-6 and Case-7 were identified as type III; based on the information at position 216, Case-1 to Case-5 might be type II or type III, whereas Case-6 and Case-7 might be type I or type II. Combining the information at these two positions, it is concluded that Case-1 to Case-5 might be type II or type III, whereas Case-6 and Case-7 are type III.

As shown by the BLAST analysis of the IS900 gene, DMC gene, and IS1311 gene, Case-1 to Case-5 might be type II, whereas Case-6 and Case-7 might be type III. Because the F57 gene is highly conserved, it can be only used to identify MAP but not the strain type of MAP. Furthermore, the 16S rRNA gene cannot be used to differentiate closely related species or different strains within the same species [34]. Therefore, this gene can only be used to identify MAC but not a particular MAC species.

In summary, Case-1 to Case-5 were identified as type II, whereas Case-6 and Case-7 were identified as type III (Table 5). In other words, the sheep and goats in Inner Mongolia are likely affected by both type II and type III MAP. Specifically, type II MAP is present in the western and eastern regions of Inner Mongolia and type III MAP is present in the western and central regions. Although it is was not surprising to find that sheep and goat herds in Inner Mongolia are infected with MAP, it was somewhat surprising to find two genetic types of MAP in these herds.

**Table 5:** Results of PCR typing, BLAST, and sequence analysis of each pathological sample in this study.

Case No.	Result					Final results
	F57 PCR + BLAST	16S rRNA PCR + BLAST	DMC PCR + BLAST	IS1311 PCR + Enzyme digestion + Sequence analysis + BLAST	IS900 PCR + Sequence analysis + BLAST + Evolutionary tree	
1	+, MAP	+, N	C, II	+, C, B/C, II	+, II/III, II, II	II
2	+, MAP	+, N	C, II	+, C, B/C, II	+, II/III, II, II	II
3	+, MAP	+, N	C, II	+, C, B/C, II	+, II/III, II, II	II
4	+, MAP	+, N	C, II	+, C, B/C, II	+, II/III, II, II	II
5	+, MAP	+, N	C, II	+, C, B/C, II	+, II/III, II, II	II
6	+, MAP	+, N	S, III	+, S, S/C, III	+, III, III, III	III
7	+, MAP	+, N	S, III	+, S, S/C, III	+, III, III, III	III

Note: N stands for uncertainty

Different strains types of MAP have been reported in different counties [28], for example, type C strain in sheep in United Kingdom and Australia; type S in sheep and goats in New Zealand, Australia, Canada, and Norway as well as goats in the Czech Republic and sheep in the Faroe Islands, South Africa, and Morocco. Sheep in Spain have been found to be mainly infected with type S strains, whereas goats are infected with both type S and type C strains. Furthermore, the type S strain has been considered as the primary genetic type that infects sheep and goats in Cyprus [35]. It is generally believed that ovine paratuberculosis is mostly caused by type S strains of MAP in sheep. In contrast, caprine paratuberculosis may be caused by goat-adapted type C strains of MAP in goats [36]. In practice, the naming convention of types S and C does not involve any host-species specificity. Both type S and type C can infect multiple animal species, including wildlife species and non-ruminants, and can even be transmitted between different species [3, 37]. It has already been shown that the same MAP strain can infect wildlife species and two species of farmed ruminants on the same farm [37].

It is unsurprising to find the presence of multiple MAP types in the same country or region. For example, type III, type B, and type II MAP have all been found in Canada [20]. Few data sets exist that combine information on the MAP genotype and the outcome or severity of disease in the field, and virulence has mostly been determined for laboratory-cultured strains using animal models. There appears to be differences between type S and type C strains with respect to their virulence in different host species [37]. Therefore, further analysis of the strain virulence of MAP in sheep and goats in Inner Mongolia is required to help lay the foundation for paratuberculosis prevention and control in the local region.

MAP or MAP DNA have been found in the blood or infected intestinal tissue taken from some patients with Crohn’s disease [38]. MAP has also been isolated from the stool samples of healthy humans and those infected with unrelated diseases [39]. Therefore, the significance of MAP as a zoonotic pathogen remains unclear [26, 40]. However, the isolates collected from the Crohn’s patients in the same country often show a close relation with each other. In addition, these isolates were also found to be related to the MAP isolates from sheep and cattle in the same country. The combined evidence suggests that livestock may be the source of transmission [3, 41]. Furthermore, MAP has been considered a risk factor for individuals with a genetic susceptibility to autoimmune diseases, such as type I diabetes, sarcoidosis, multiple sclerosis, and Hashimoto’s thyroiditis [42]. It has been confirmed that MAP can be easily isolated from the milk (including pasteurized milk) or muscle tissue of infected cattle [43–45]. Therefore, the monitoring, prevention, and control of paratuberculosis in animals should receive significant attention in all countries globally.

## Conclusions

It was confirmed in this study for the first time that sheep and goats in Inner Mongolia, China, were infected with two subtypes of MAP strains. The results obtained in this study have significant epidemiological implications for the control and prevention of paratuberculosis in China.

## Supporting information

S1 Fig (JPG)

S2 Fig (JPG)

S3 Fig (JPG)

S4 Fig (JPG)

S5 Fig (JPG)

S6 Fig (JPG)

S7 Fig (JPG)

S8 Fig (PDF)

## Abbreviations

MAA: *M*. *avium* subsp. *avium*
MAC: *Mycobacterium avium* complex
MAH: *M*. *avium* subsp. *hominissuis*
MAP: *Mycobacterium avium* subsp. *paratuberculosis*
NJ: neighbor-joining
PCR: polymerase chain reaction
REA: restriction endonuclease analysis
