# Supplementary figures and images for: Serological investigation and genotyping of Mycobacterium avium subsp. paratuberculosis in sheep and goats in Inner Mongolia, China
Source: PLoS One. 2021 Sep 7;16(9):e0256628. doi: 10.1371/journal.pone.0256628 (PMC8423245; doi:10.1371/journal.pone.0256628)

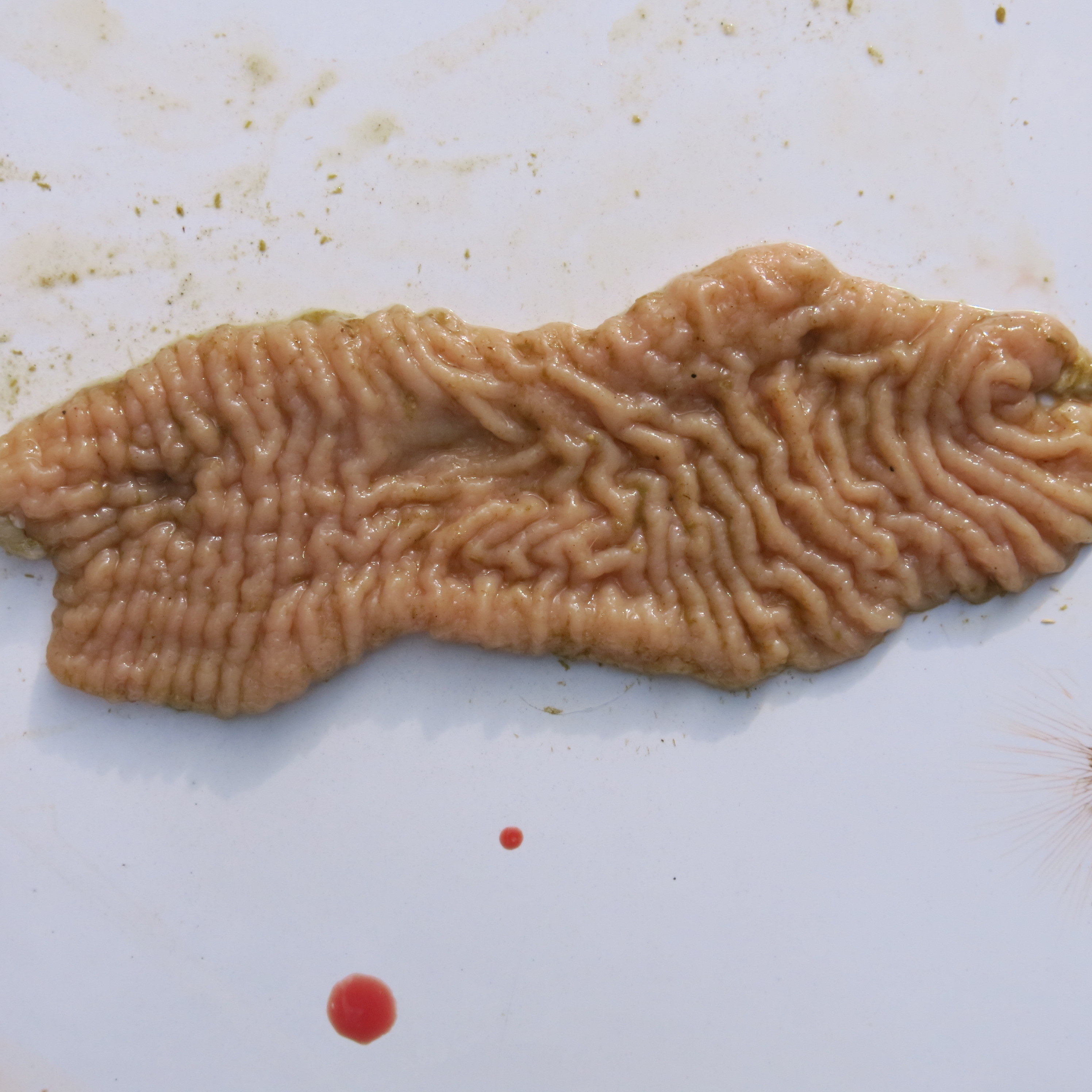

Supplement: S1 Fig — (JPG) [file pone.0256628.s001.jpg]

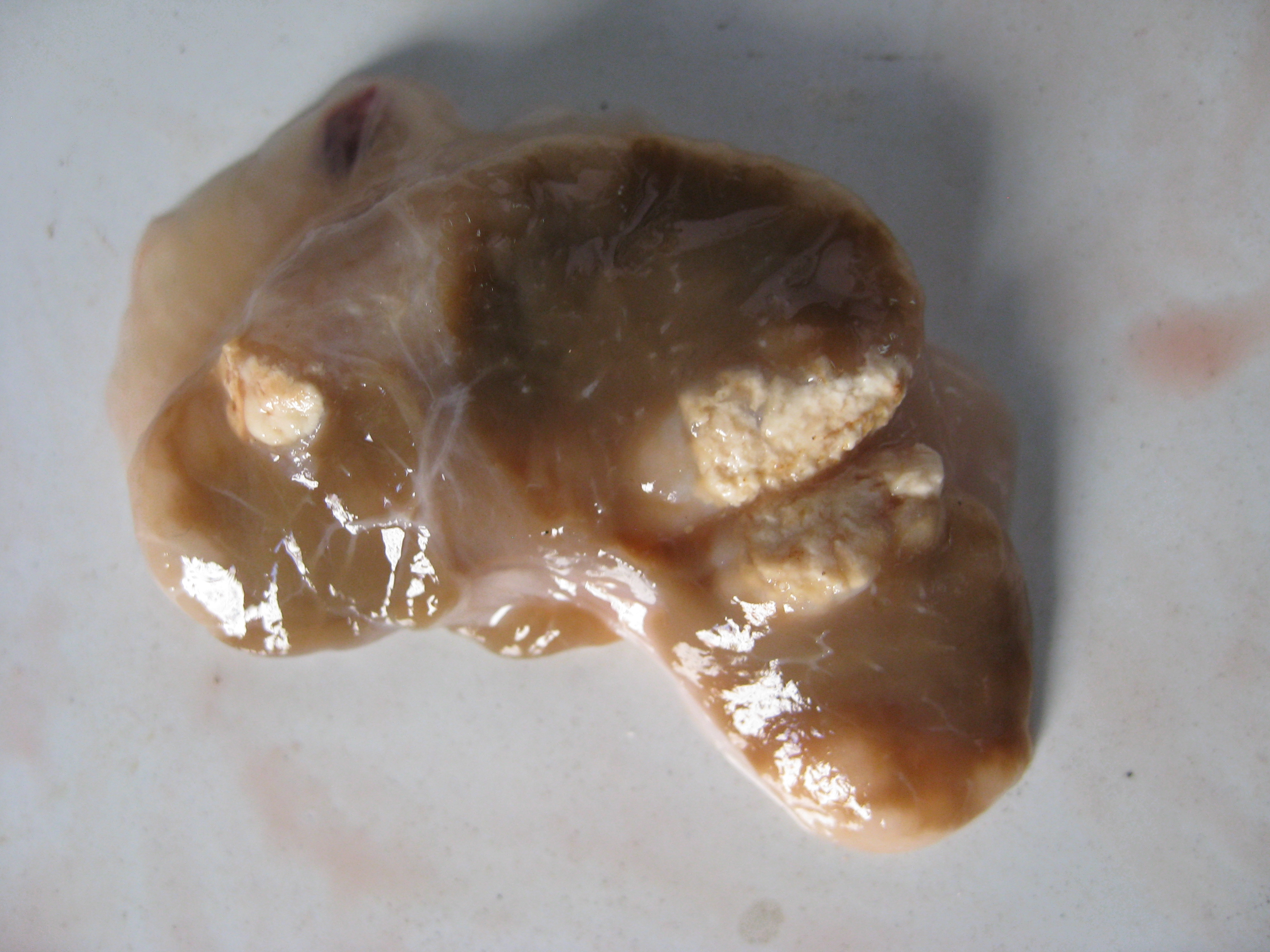

Supplement: S2 Fig — (JPG) [file pone.0256628.s002.jpg]

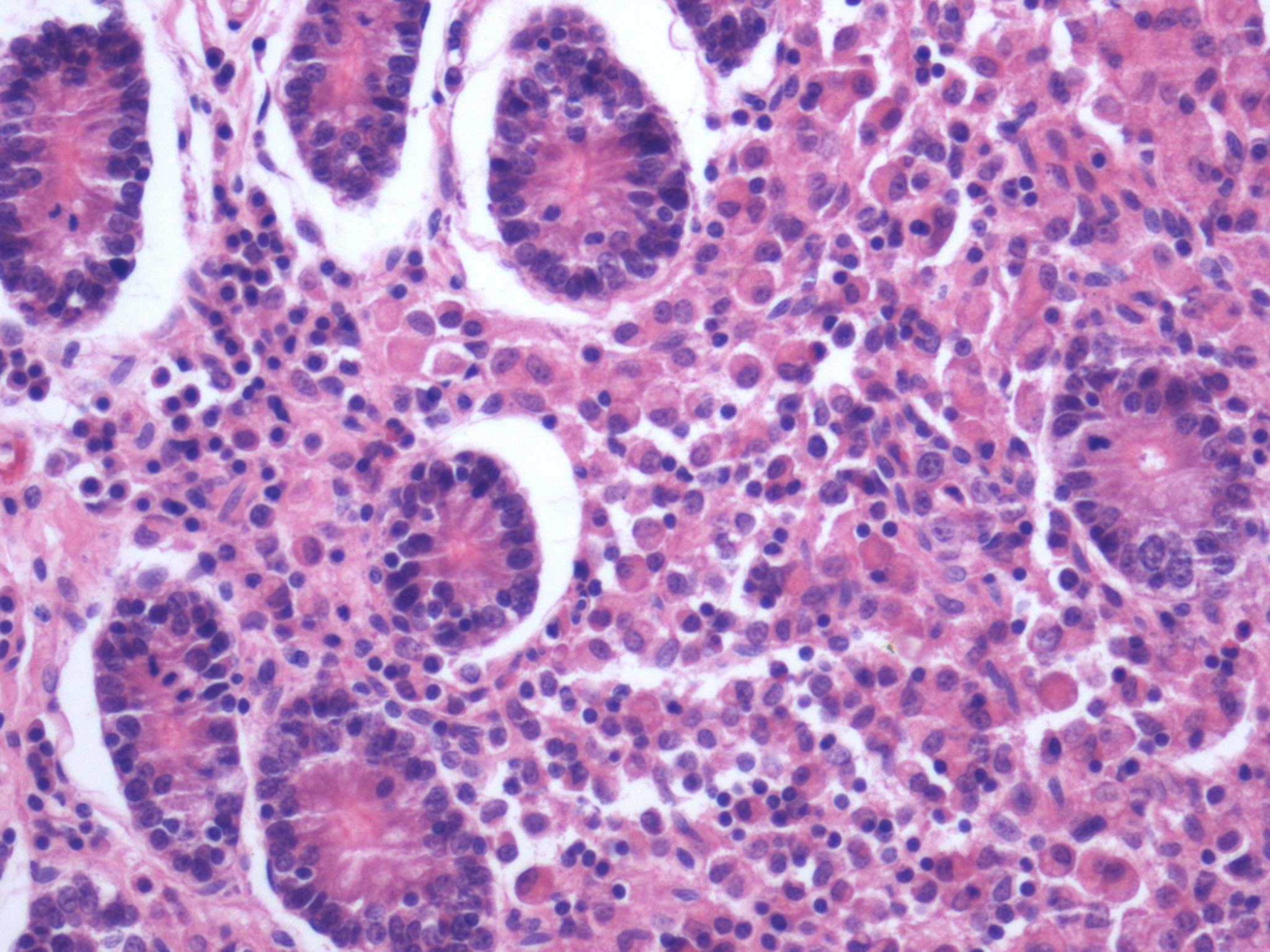

Supplement: S3 Fig — (JPG) [file pone.0256628.s003.jpg]

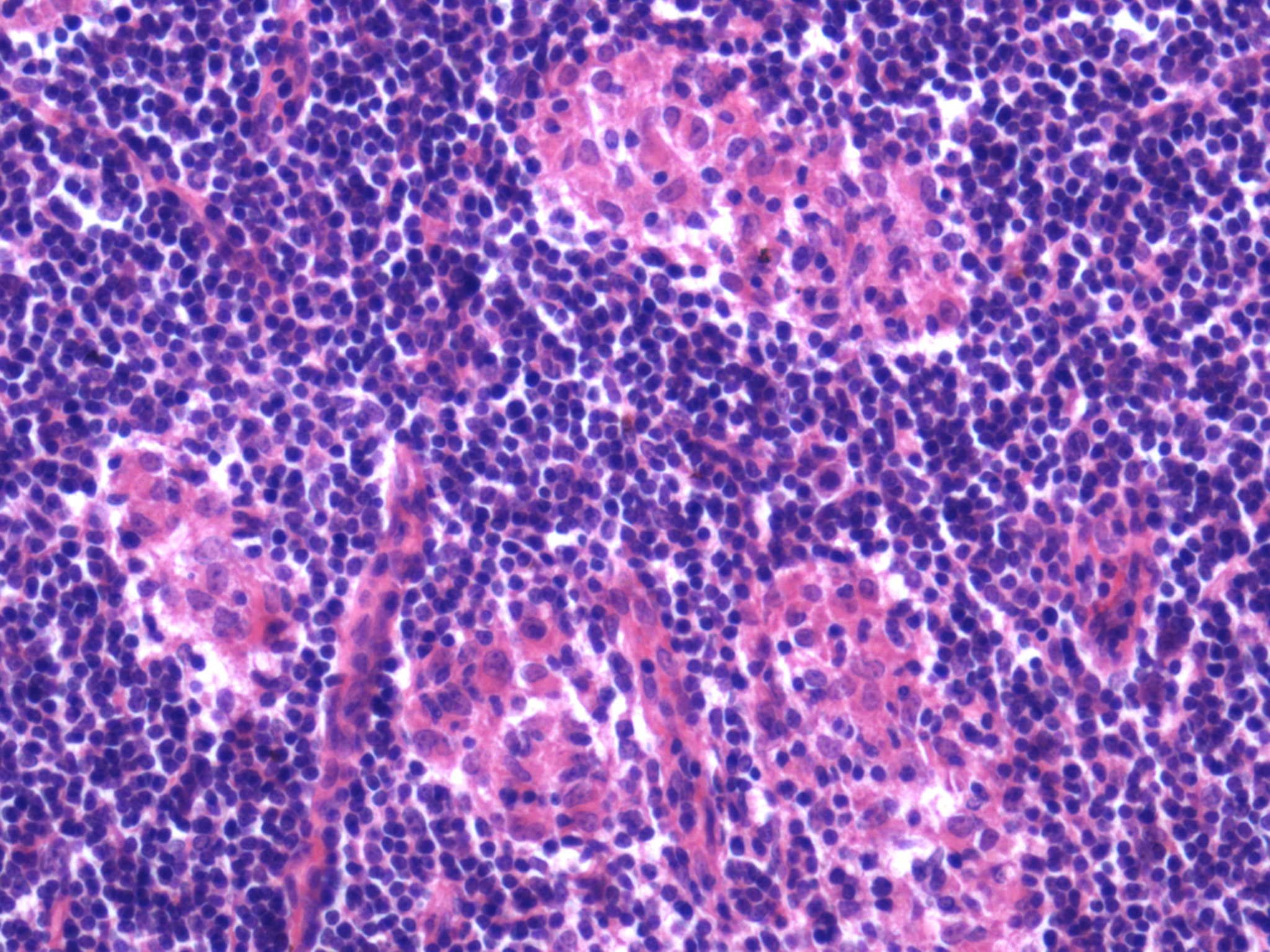

Supplement: S4 Fig — (JPG) [file pone.0256628.s004.jpg]

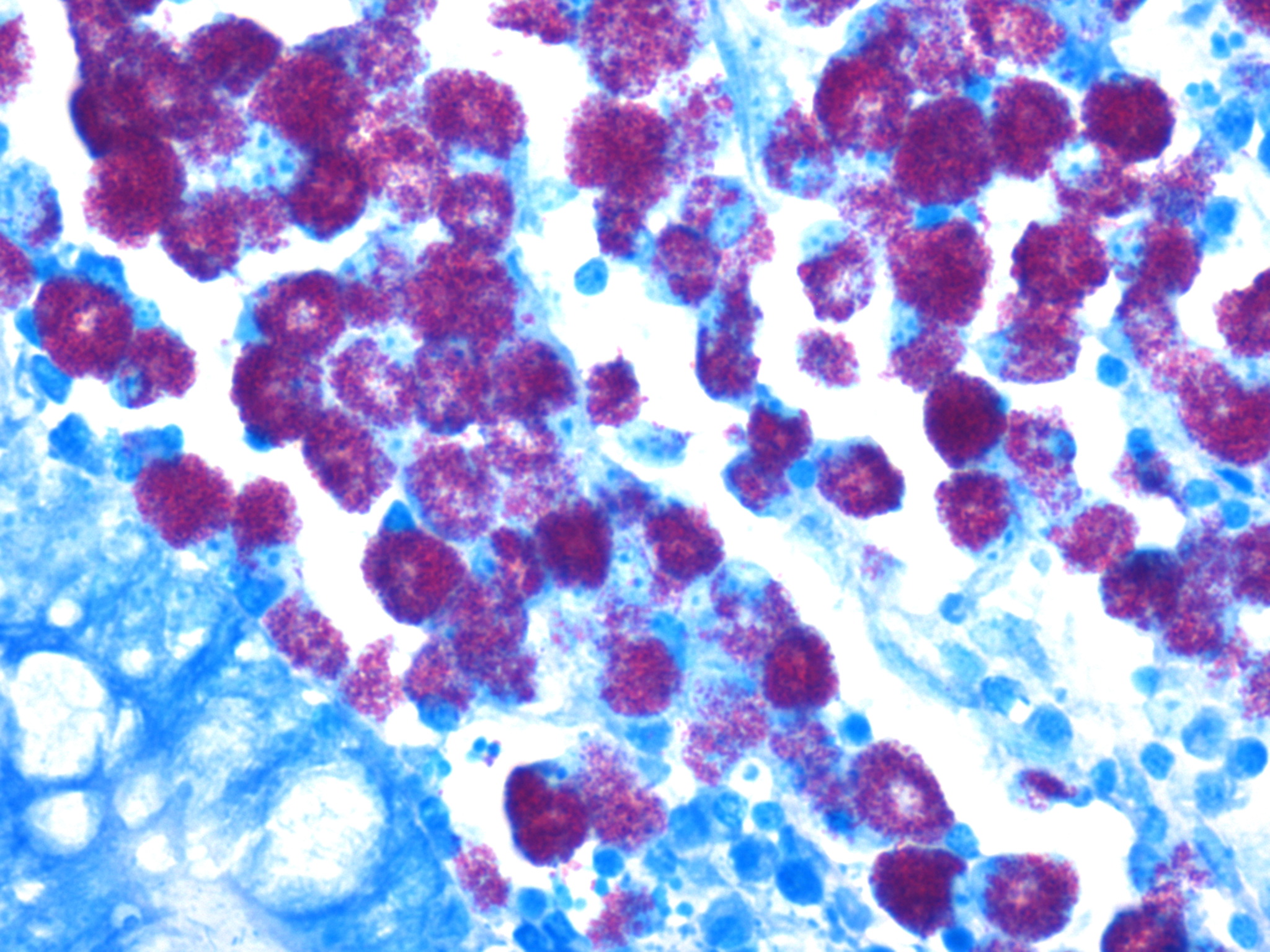

Supplement: S5 Fig — (JPG) [file pone.0256628.s005.jpg]

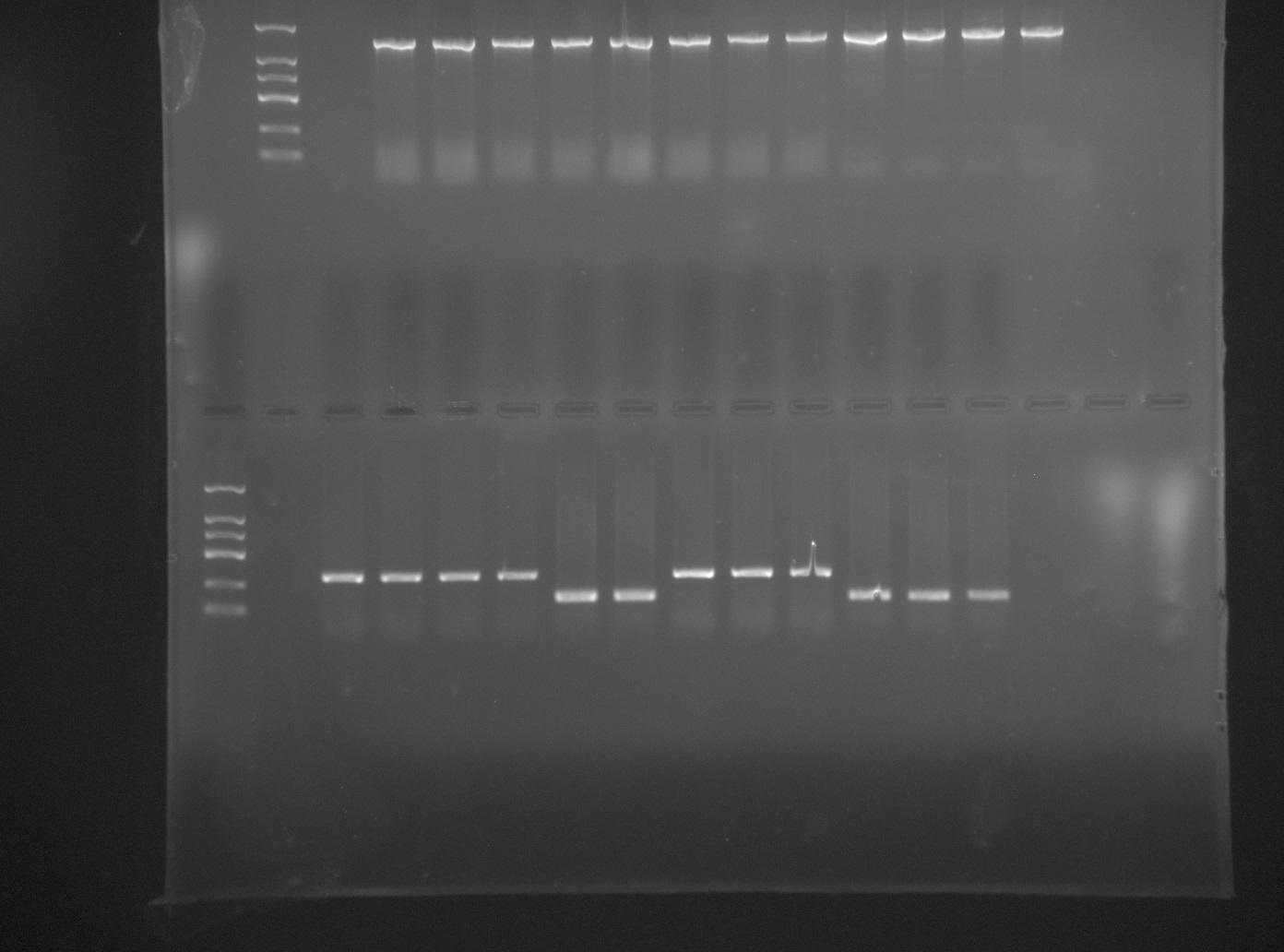

Supplement: S6 Fig — (JPG) [file pone.0256628.s006.jpg]

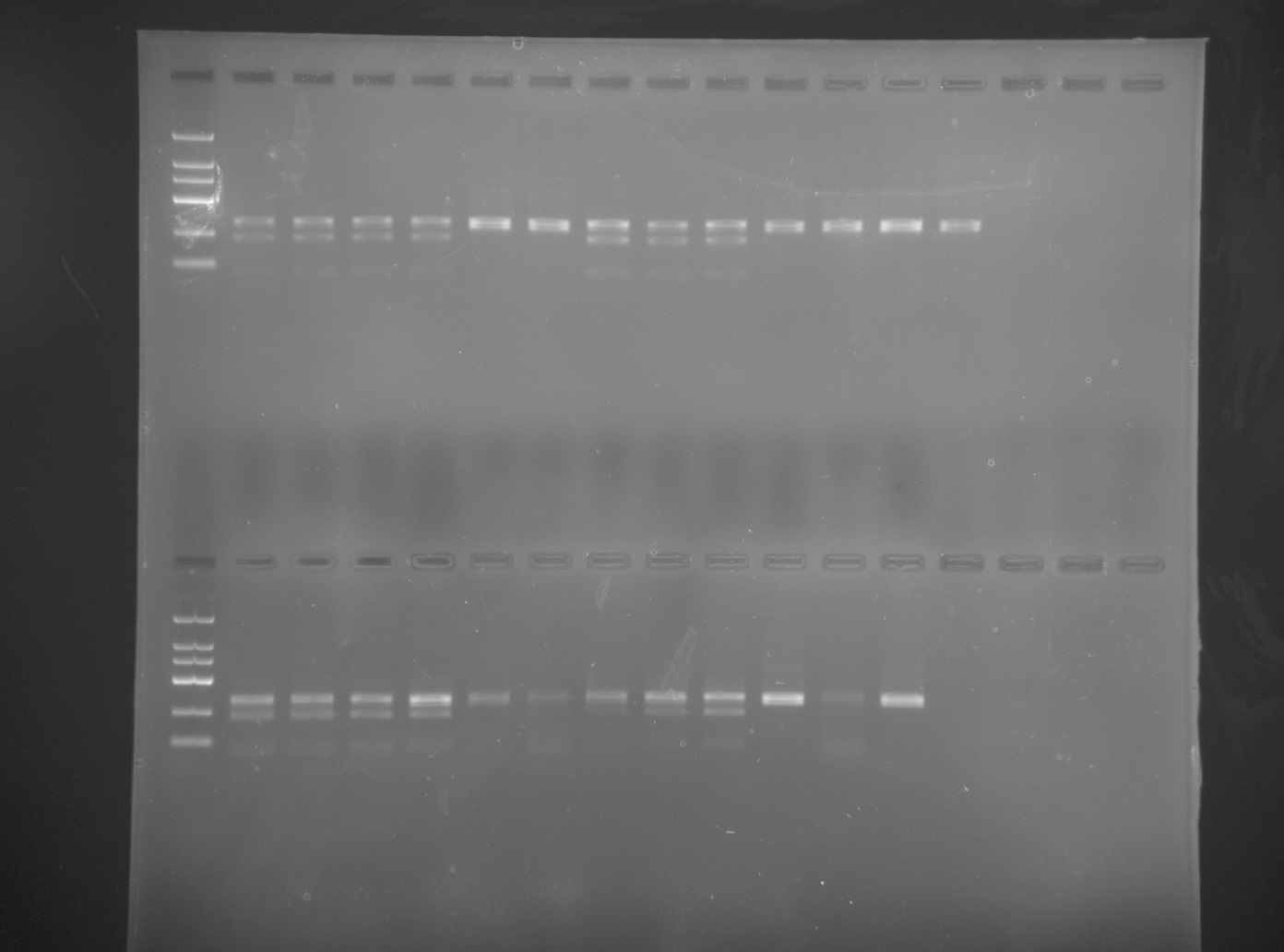

Supplement: S7 Fig — (JPG) [file pone.0256628.s007.jpg]

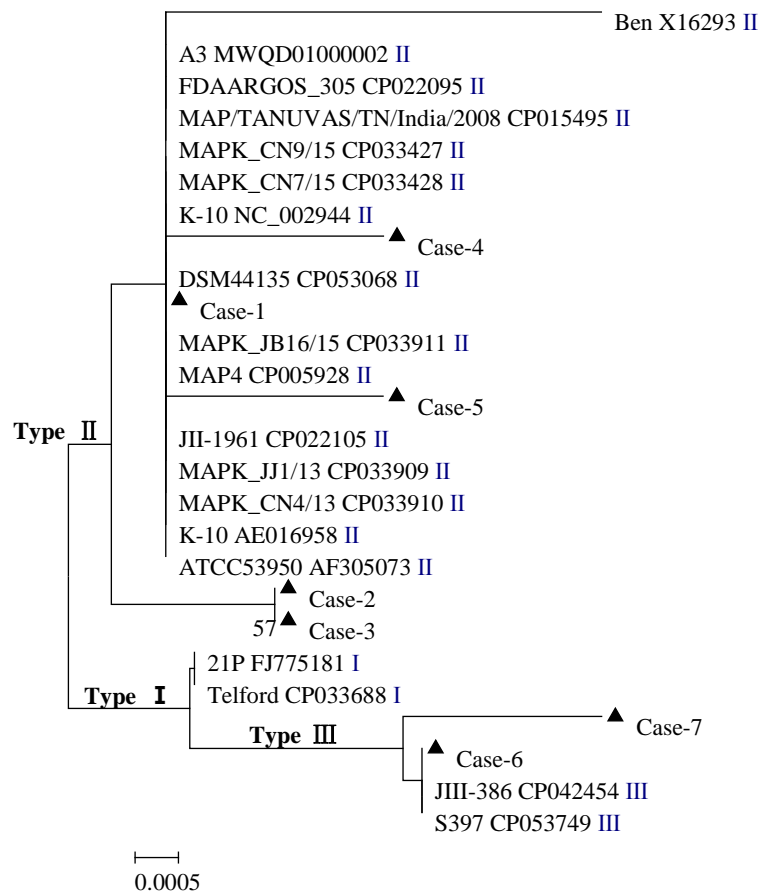

Supplement: S8 Fig — (PDF) [file pone.0256628.s008.pdf]
